# Effect of Psychotherapy on Reduction of Fear of Childbirth and Pregnancy Stress: A Randomized Controlled Trial

**DOI:** 10.3389/fpsyg.2020.00787

**Published:** 2020-05-26

**Authors:** Somayeh Abdollahi, Mahbobeh Faramarzi, Mouloud Agajani Delavar, Fatemeh Bakouei, Mohammad Chehrazi, Hemmat Gholinia

**Affiliations:** ^1^Counseling in Midwifery, Student Research Committee, Babol University of Medical Sciences, Babol, Iran; ^2^Department of Biostatistics and Epidemiology, School of Public Health, Babol University of Medical Sciences, Babol, Iran; ^3^Community Health, Infertility and Reproductive Health Research Center, Health Research Institute, Babol University of Medical Sciences, Babol, Iran; ^4^Reproductive Health, Infertility and Reproductive Health Research Center, Health Research Institute, Babol University of Medical Sciences, Babol, Iran; ^5^Department of Epidemiology & Biostatics, Babol University of Medical Sciences, Babol, Iran; ^6^Biostatistics, Social Determinants of Health Research Center, Health Research Institute, Babol University of Medical Sciences, Babol, Iran

**Keywords:** fear of childbirth, psychotherapy, pregnancy-specific stress, anxiety, motivational interviewing, self-efficacy

## Abstract

**Introduction:**

The fear of childbirth (FOC) has an adverse effect on the physical and mental health of pregnant women and increases adverse maternal and fetal outcomes. Previous research reported the effect of psychological interventions such as cognitive behavioral therapy, relaxation therapies, and short-term psycho-educational intervention on FOC. We examined whether adding motivational interviewing (MI) psychotherapy to prenatal usual care (PUC) is superior to PUC alone to reduce the scores of FOC, pregnancy stress, and self-efficacy.

**Materials and Methods:**

An RCT with two-arm parallel groups and 1:1 allocation ratio assigned 70 pregnant women (aged 18–50) attending public health centers in an education hospital in Iran to receive five sessions of group MI psychotherapy plus PUC (*N* = 35) or to receive PUC alone (*N* = 35). The primary outcomes were the FOC scores (Wijma Delivery Expectancy/Experience Questionnaire, W-DEQ), pregnancy-specific stress (Prenatal Distress Questionnaire, NuPDQ), anxiety (Spielberger state anxiety), and Childbirth Self-Efficacy Index (CBSI) at 5 weeks post-randomization. Additional measures included subscales of the W-DEQ and the NuPDQ, patients’ compliance, and satisfaction with psychotherapy intervention at 5 weeks post-randomization as secondary outcomes.

**Main Results:**

The post-trial results indicated that the outcome scores diminished more considerably in psychotherapy than in PUC for total FOC scale with a large effect size (*B* = −23.54, *p* = < 0.001, η^2^η^2^ = 0.27), for total pregnancy stress with a large effect size (*B* = −4.51, *p* = < 0.001, η^2^ = 19), and for state anxiety with a large effect size (*B* = −12.42, *p* = < 0.001, η^2^ = 0.22). However, the score of self-efficacy and concern about physical symptoms did not differ between the psychotherapy and PUC groups (*P* < 0.05).

**Discussion:**

Adding 5 weeks of group psychotherapy to PUC could be considered as an adjunctive care option for reducing FOC, pregnancy stress, and general anxiety in pregnant women in the third trimester. Future research may focus on sustaining the effects and evaluating the economic impacts of adding psychotherapy to PUC.

## Introduction

Around 80% of pregnant women experience worries and fears in relation to their upcoming childbirth (Melender, 2002). The fear of childbirth (FOC) is defined as an unreasonable dread of childbirth. To date, no clear specific definition has been offered for FOC or what levels may constitute a phobic response (Slade et al., 2019). FOC is commonly framed as issues associated with expression of anxiety and stress, while others have defined it as a specific construct. Some evidence supports FOC as a symptomatic concept. There are associations between FOC and psychiatric disorders such as mood and anxiety disorders, PTSD, and personality disorders (Rouhe et al., 2011). Another piece of evidence is the relationship between physical/sexual abuse or trauma and FOC (Kjærgaard et al., 2008). Also, the literature has classified FOC into primary and secondary. Primary FOC is described as FOC in nulliparous women. Psychosocial aspects of life and experiences of trauma and abuse are associated with primary FOC. Secondary FOC is defined as the FOC after traumatic birth (Hofberg and Ward, 2003). Nevertheless, the distinction between primary and secondary FOC is very difficult. Meanwhile, a possible vicious circle has been suggested in FOC. Women with high levels of FOC during the antenatal period might experience more fear during labor and delivery. Hence, FOC in nulliparous women is a result of complex etiologies such as general anxiety/depression and previous abuse (Barlow and Durand, 2015). Thus, a biopsychosocial approach may help in presenting a definite classification and the etiology of FOC (Rondung et al., 2016).

The FOC has a negative effect on the emotional health of pregnant women and increases the likelihood of childbirth becoming an undesirable experience (Haines et al., 2012). It ranges from mild (phobic thoughts about parturition) to severe (tokophobia) with severe distress and avoidance thoughts and behaviors in pregnant women. The prevalence of FOC has been reported to be different across various cultures, definitions, and countries. Approximately 3.5–49% of pregnant women are afraid of childbirth (O’Connell et al., 2017). Studies have shown that FOC is one of the causes of elective cesarean section (Okonkwo et al., 2012). This condition ranges from rational to irrational fear (Salomonsson et al., 2010). It may also increase the risk of psychological problems (Rouhe et al., 2011; Rondung et al., 2016) as well as unnecessary cesarean section (Vladic, 2006). Sometimes, the FOC leads to avoiding pregnancy and motherhood or denial of pregnancy (Nilsson and Landgren, 2009).

Pregnancy stress is categorized into two types: general stress and pregnancy-specific stress (PSS). PSS refers to the concerns and worries of the mother associated with the pregnancy, including labor and childbirth, physical symptoms of parenting, relationship with others, and fetal health (Faramarzi and Pasha, 2018). Negative feelings about body appearance, hormonal changes, and the new role of motherhood may exacerbate the pregnancy stress (Osman et al., 2010). Evidence suggests that fetal health has been a major concern for mothers (Georgsson Ohman et al., 2004). Most pregnant women have anxiety about screening tests for fetal abnormalities (Kleinveld, 2008). Several factors affect the severity of experience of PSS, such as stress coping strategies and women’s social support, as well as lifestyle (Faramarzi and Pasha, 2015; Faramarzi et al., 2016; Omidvar et al., 2018). Maternal stress during pregnancy causes low birth weight, preterm labor, and prolonged labor pain, along with behavioral and emotional disorders in children (Relier, 2001; O’Connor et al., 2002; Simpson and Creehan, 2003; Jannati and Khaki, 2005). Recent evidence has indicated that PSS is a stronger predictor of birth outcomes as compared with general stress (Dunkel Schetter and Tanner, 2012; Hasanjanzadeh and Faramarzi, 2017). PSS is associated with an increased risk of pregnancy complications, including preeclampsia, abortion, and the severity of the disease (Faramarzi et al., 2015, 2019; Haghparast et al., 2016). There is a relationship between maternal stress and FOC (Klabbers et al., 2016). Indeed, a recent study reported that PSS was moderately and positively correlated with FOC (Kabukcu et al., 2019). Specifically, common concerns of women in the third trimester are the birth, labor pain, and perianal tearing (Pasha et al., 2012; Fenwick et al., 2015).

Self-efficacy of pregnant women is defined as the assessment of their ability to cope with stressful situations, including labor time, and to perform essential behaviors at times of stress. Pregnant women with high self-efficacy experience a lower level of fear and pain and thus have more satisfaction with childbirth. Conversely, a low-self-efficacy pregnant woman has intense fears during pregnancy and finds it impossible to undergo a normal childbirth (Salomonsson et al., 2013b). There is a relationship between FOC and stress, self-efficacy, and choice of natural delivery (Toohill et al., 2014). Women with high levels of self-efficacy have more capability to cope with perinatal stress as well as childbirth (Salomonsson et al., 2013b). Evidence has emphasized self-efficacy expectancy of pregnant women as negatively correlated with FOC (Lowe, 2000).

Although the evidence base for the effect of psychological interventions for improving pregnancy stress is robust (Asghari et al., 2016; Zhang et al., 2019), to the best of our knowledge, no randomized clinical trial study has investigated the effect of motivational interviewing (MI) psychotherapy for reducing the FOC. Nevertheless, there is some evidence to support the potential effectiveness of MI psychotherapy on FOC. Initially, MI psychotherapy promotes behavioral changes and provides a framework which facilitates lifestyle changes through resolving ambivalences (Arkowitz et al., 2008; Ghahari, 2013; Salomonsson et al., 2013a). The beneficial health outcomes of MI psychotherapy have been widely endorsed (Martins and Mcneil, 2009; Bahmani et al., 2019; Motahari Tabari et al., 2019). Secondly, the core of the effectiveness of MI psychotherapy is breaking the clients’ resistance to change by resolving ambiguities. Indeed, some evidence also suggests that part of the stress and fears of childbirth is rooted in ambivalence (contradictory emotions) about the maternal role (Brid and Wendy, 2002). Further, previous research has shown that MI is effective on anxiety (Westra et al., 2009). Finally, evidence also supports that motivational counseling psychotherapy enhances clients’ self-efficacy (Walpole et al., 2013).

To address these gaps in the literature, we designed a study to assess the effectiveness of psychotherapy on the FOC and pregnancy stress. To the best of our knowledge, this is the first randomized clinical trial designed to investigate the effect of psychotherapy for improvement of FOC, pregnancy stress (both general stress and PSS), and self-efficacy. The primary aim of the study was to determine whether adding MI psychotherapy to prenatal usual care (PUC) is superior to PUC alone to reduce the scores of FOC, pregnancy stress, and self-efficacy. The secondary aim was to test the patients’ compliance and satisfaction with adding MI psychotherapy to PUC.

## Materials and Methods

### Study Design

This randomized controlled trial was a two-arm parallel-group study with a 1:1 allocation ratio to either MI psychotherapy plus PUC (experimental group) for pregnant women in an antenatal clinic of an educational hospital or PUC alone (control group).

### Participants

This study was conducted in Yahyanejad Educational Hospital of Babol University of Medical Sciences from September to December 2018. Women who were referred to obstetrics clinic of the center were recruited for this study.

Eligibility criteria included gestational age of 26–33 weeks, at least 5 years of primary education, 18–50 years of age, no previous cesarean section delivery, not undergoing psychotherapy until 5 weeks afterward, and no complicated pregnancy. The participants were excluded if they reported a history of schizophrenia, bipolar disorder, severe depression, or suicide; were currently on any psychotherapy or antidepressant medications; or had a complicated pregnancy such as hemorrhage, hypertension, diabetes, and any indication for termination of pregnancy.

A member of the group research team (first author) invited the women to enter the study. She attended prenatal education classes that were delivered for the pregnant women in a small group, 1 h every week, with a midwife in the obstetrics clinic. During the last 20 min of the class, the first author had a lecture about the fear of birth, signs, complications, and strategies for reducing it. She explained that the research team was conducting a psychological intervention to reduce the FOC and pregnancy stress with a psychologist. Also, she introduced the goals, interventions, and duration of the study, and invited pregnant women to participate in the study. At the end of the lecture, she answered the questions of the pregnant women. After the class, she assessed the inclusion criteria for volunteer pregnant women who accepted the invitation to enter the study. If the pregnant women met the inclusion criteria, they were enrolled in the trial and referred to a midwife outside the research team. The expert midwife gave them demographic and pretest questionnaires. Also, all participants completed the informed consent form.

**FIGURE 1:**
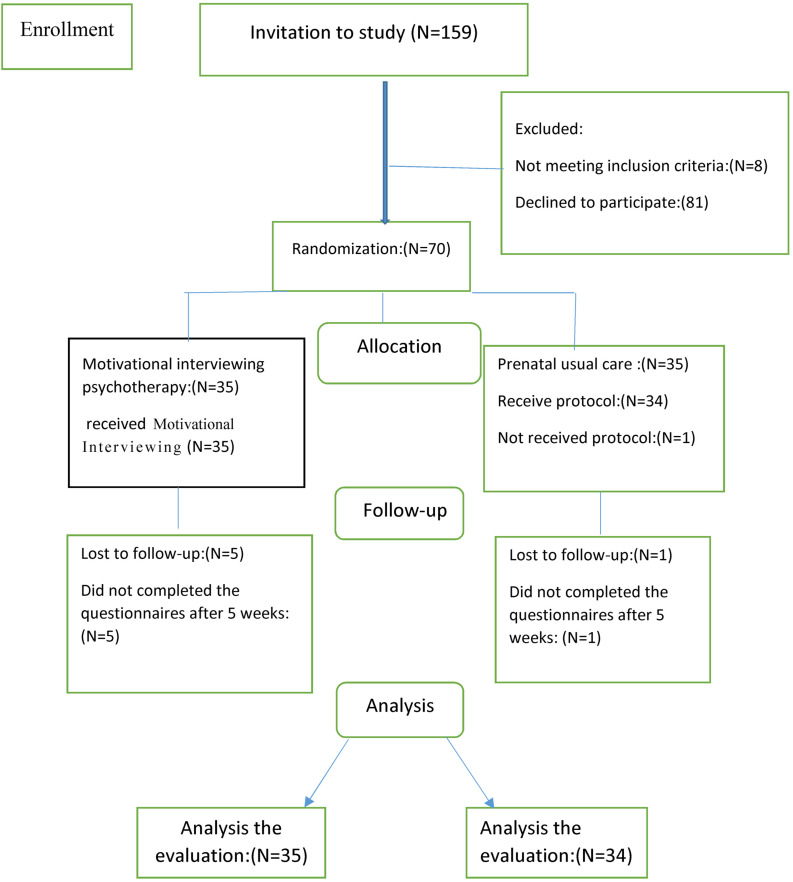
Consort flow diagram of the participants.

A total of 159 pregnant women over 23 weeks of gestation were invited to participate in the study. Specifically, 89 of them either declined the invitation or did not meet the inclusion criteria. Overall, 70 eligible individuals were randomly assigned into control (Toohill et al., 2014) and intervention (Toohill et al., 2014) groups. Of women who completed the pre-trial questionnaire in the psychotherapy group, five withdrew before the post-trial. Of 35 women randomized to PUC, 1 person withdrew after randomization, as she did not complete her pre-trial questionnaires. Of 34 women who completed the pre-trial questionnaire, 100% provided data at post-trial (Figure 1).

### Sample Size

Sampling was performed as available sampling on pregnant women who were referred to the obstetrics clinic of the hospital. As there was no research outlining the efficacy of MI on FOC, pregnancy stress, or childbirth efficacy in pregnant women, the power calculation was informed by published RCTs of CBT for reducing FOC in pregnant women and MI psychotherapy for enhancing self-efficacy in non-pregnant women (Bahmani et al., 2019; Ghazaie et al., 2019). Furthermore, to calculate the sample size, we considered the mean difference of FOC and childbirth self-efficacy between the two intervention groups. To detect a between-group effect corresponding to a Hedges g of 2.18, the minimum sample size for each group (α = 0.05, power of 80%) was 31 participants per group. Thus, we aimed to recruit a minimum sample of 35 participants per group to allow for expected attrition.

### Randomization and Blinding

An independent midwife completed the randomization according to a 1:1 ratio within blocks of four (balanced permuted block randomization) using a random number computer generator^1^. Given the two groups of 4 blocks, with 70 participants, 18 random blocks were generated by the computer (the last 2 blocks were used). The allocation sequences were kept and were not made available to any of the patients or researchers.

The outcome assessments during pre-trial and post-trial were conducted by a staff member who was unaware of the treatment allocation and not involved in the recruitment of the women. The statistical analyzer did not know the coding of intervention or control groups either.

### Interventions

#### Motivational Interviewing Psychotherapy

Women allocated to the experimental treatment received MI psychotherapy, introduced by Miller and Rollnick (2009), plus PUC. The MI approach is client-centered, which provides the atmosphere for a natural change and allows the client to explore and solve ambivalences. MI consists of three components: collaborative spirit, evocation, and respect for the patient’s autonomy. It includes five specific enhancement techniques which can be used by an MI therapist to address the needs of the clients. The enhancement techniques include: open questions, establishing a schedule with the patient, reflective listening, stimulating the plan of action, and summarizing the conversation. Also, the MI therapist has some important skills including empathy, avoiding arguments, talking about behavior change, drawing attention to discrepancies, respecting autonomy, empowering the patient, and a joint decision-making process (Miller and Rollnick, 2009).

MI psychotherapy was conducted by a female therapist (author MF) through weekly 120 min face-to-face group sessions for a period of 5 weeks. The therapist had enough experience in the MI approach. A female assistant (author SA), who was trained in the MI approach before the trial, helped the therapist in the sessions. The assistant taught the women through group work and guided exercise. To consolidate their knowledge, the participants were given home exercises and were encouraged to write their exercises on a daily record sheet. At the beginning of each session, the assistant asked the participants to describe their individual experience of FOC outside of the class. Also, the assistant helped the participants to document the corrected MI skills.

The MI psychotherapy consisted of five main modules which were based on the FOC and pregnancy stress. The first MI session focused on understanding the stages of change based on the Prochaska and DiClemente model (1999) for FOC and pregnancy stress. The second session involved paying attention to maternal feelings and ambivalences. The third session emphasized the positive and negative aspects of behavior change. The fourth session focused on the realization *values*. Finally, in the fifth session, the participants learned to identify dangerous situations and tempting recurrence of childbirth fears. The therapist-resolved the ambivalent behavior regarding FOC or pregnancy stress through five steps: (1) assessing the client’s motivation and confidence on subjective scales of 1–10, to observe motivation for modifying the ambivalent behaviors; (2) detecting facilitators for changing ambivalent behaviors; (3) eliciting the “pros” and “cons” of any stress or fear; (4) providing a menu of options to address any barriers; and (5) assessing the client’s values and goals to resolve ambivalence between the current behaviors and goals/values (Miller and Rollnick, 2009; Ghazaie et al., 2019).

During the sessions, the therapist used the MI techniques to deliver the intervention, which guided pregnant women on applying the MI model to cope with their fears and stress. She also encouraged them to complete exercises and review the assignments with key learning points handed out after each session. The outlines of the five sessions are presented in Table 1.

**TABLE 1:** Outline of the motivational interviewing sessions for the fear of childbirth and pregnancy stress in pregnant women.

Session 1: Stages of change
Focus on setting strategies, stress related to pregnancy, building a therapeutic alliance, building motivation to change her behavior about the fear of childbirth, and an introduction to MI in-session practice.
Assignment: Assess the *stage of change* of the client based on Prochaska and DiClemente model (1999) for the ear of childbirth and pregnancy stress. Definitions of causes of anxiety and fear of childbirth, the importance of coping with pain, anxiety and fear of childbirth. Assess the client’s motivation and confidence on subjective scales of 1–10. Freedom Exercise, Behavioral impact dimension exercise, Commitment and assessment.
Worksheets for home assignment: Attention to her stages of change to cope with the fear of childbirth and pregnancy stress.
Session 2: Maternal feelings and ambivalences
Reflection on the last week and repetition.
Helping the client to express her feelings regarding the pregnancy stress and fear of childbirth. Helping the client to recognize her ambivalences regarding the pregnancy stress and fear of childbirth.
Assignment: Attention to her negative and positive feelings; Attention to ambivalences regarding the vaginal delivery; detecting facilitators for changing ambivalent behaviors.
Session 3: positive and negative aspects of behavior change
Reflection on the last week and repetition.
Dealing with positive and negative aspects of behavior change regarding the fear of childbirth and pregnancy stress.
Assignment: Elicit the “pros” and “cons” of any stress or fear regarding the pregnancy or childbirth.
Session 4: values and goals
Reflection on the last week and repetition.
Assignment: Provide a menu of important values to address any barriers for changing the ambivalences.
Provide a list of values in terms of personal importance and select around five that are most important. Discuss why the values/goals selected are important to them. To explore what connections they see between their current pregnancy stress and fear of childbirth and their ability to achieve these goals or live out these values.
Session 5: Dangerous situations and tempting return
Reflection on the last week and repetition. Helping the client identify the dangerous situations and tempting return of childbirth fears. Helping the patient to develop a practice of her own, review the progress, insights, techniques, and the individual evaluation of the sessions. Reflection on the learned skills and final discussion. Assignment: To deal with the dangerous situations and tempting return of pregnancy stress and fear of childbirth.

For individual practice, the participants received printed copies of the material about an important part of the program and homework assignment (i.e., stages of change); they performed daily formal practice for 30 min per day over a period of 5 weeks.

### Control Group

Women allocated to the control group received PUC by a clinician in the hospital. The women completed the questionnaires at the beginning of the trial and 5 weeks post-trial. The clinician (almost a midwife) provided prenatal care for pregnant women based on prenatal guidelines, “The Iranian national program on safe motherhood, integrated care on mother’s health” (Iranian Ministry of Health and Medical Education, 2010). The main components of PUC for all women in our teaching hospital included evaluations of maternal and fetal health, clinical examination, measurement of blood pressure, testing of urine and blood, education of health, and optional weight plus height measurement. Screening tests were carried out on the first trimester (6–10 weeks of pregnancy) and included screening tests, blood count, Rh test, fasting blood sugar, BUN, creatinine, venereal disease research laboratory (VDRL), urine analysis and culture, as well as a voluntary HIV screening test. Also, ultrasound examination was performed at 16–18 and 31–34 weeks of pregnancy. The midwife also provided health education on nutrition and self-care, taking vitamin supplements, iron, and folic acid. The frequency of PUC visits for an uncomplicated pregnancy was eight times at weeks 6–10, 11–15, 16–20, 21–25, 26–30, 31–34, 35–37, and 38 until the birth time. Also, the midwife in our hospital conducted group educational prenatal care for women.

### Outcomes

#### Primary Outcomes

The primary outcomes were as follows:

##### Fear of childbirth

The Wijma Delivery Expectancy/Experience Questionnaire (W-DEQ) developed by Wijma et al. (1998) was used to assess the FOC. It includes 33 items with a five-point Likert scale (0 = no to 5 = very high), with the scores ranging from 0 to 165. It has shown good validity and reliability (Cronbach’s alpha was 0.89 and 0.93, respectively) (Wijma et al., 1998). The Persian version of the scale also has good validity and reliability with six subscales, including self-efficacy, lack of positive prediction, loneliness, fear, worry for child, and loss of control (Abedi et al., 2017; Mortazani, 2017).

##### General anxiety

To assess general anxiety, 20 items of the state anxiety questionnaire were used in the Spielberger State-Trait Anxiety Questionnaire. The tool consists of 40 questions that assess four options (by no means, sometimes, generally, very much) with 20 questions for state anxiety and 20 questions for trait anxiety. The range of scores of state anxiety was 20–80. Higher scores in each component showed higher anxiety intensity. This instrument has shown high validity and reliability (Ghazaie et al., 2019). The Iranian validated version of state anxiety was used in this study (Panahi-shahri, 2002).

##### Pregnancy-specific stress

The pregnancy stress was assessed by the revised Prenatal Distress Questionnaire (NuPDQ) developed by Lobel et al. (2000). The questionnaire has 17 items assessing pregnancy stress in the three trimesters of pregnancy, separately. Nine items are repeated in the first, second, and third trimesters. Also, three items are repeated in the second and third trimesters. Further, there are five items specifically for the third trimester stress. The tool assesses the worries of women through a three-point Likert scale (0 = no, 1 = little, 2 = very). Higher scores on the NuPDQ indicate greater stress in pregnant women (Lobel et al., 2000). The subscales of this questionnaire in the third trimester include five dimensions: medical/cost problems, physical symptoms, fetal health concerns, parental concerns, and labor pain.

##### Childbirth self-efficacy inventory

Lowe (2000) designed a self-efficacy questionnaire to measure the belief or confidence of a pregnant woman about her ability to perform self-control behaviors to cope with uterine contractions (labor pain). The reliability of the questionnaire was estimated as 0.86–0.95 (Lowe, 2000). This questionnaire should be filled up after the third trimester of pregnancy. Although the scale has 62 questions, in this study, the section of expectation of birth self-efficacy in the active phase of labor (15 questions) was used. The pregnant women would reply to each question with scores ranging from 1 (very weak) to 10 (very excellent) based on the level of “confidence in performing delivery pain control behaviors.” For example, the first question on the questionnaire is, “I keep my body relaxed.” Higher scores indicate higher self-efficacy in pregnant women. Its validity and reliability in Iran have been measured by Khorsandi et al. (2008), and its Cronbach’s alpha ranged from 0.84 to 0.91 (Khorsandi et al., 2008).

#### Secondary Outcomes

The secondary outcomes were as follows:

##### Subjective clinical improvement of FOC

The participants rated experiences of FOC through a 10-point Likert scale from 1 to 10 (1 = little to 10 = very much) before and after the treatment: To what extent do you have FOC?

##### Treatment compliance

We had three criteria for treatment compliance: the mean number of psychotherapy sessions the participants attended, how many participants provided post-treatment data, and assignment activity. The psychotherapist assessed the quality of activity of the patients based on performing the assignment during the week from 1 = weak quality to 5 = good quality.

##### Treatment satisfaction

The participants rated their satisfaction with the MI psychotherapy through a five-point Likert scale from 1 (very low satisfaction) to 5 (very high satisfaction): How much were you satisfied with the psychotherapy sessions?

### Data Analysis

All analyses were conducted using STATA. For comparing the two groups at the baseline, we used chi-square tests for categorical data and *Student’s t-tests* for continuous data. To deal with the protocol deviation, intention to treat analysis was employed for each outcome. We applied a multiple regression approach for analysis of covariance (ANCOVA) to estimate the differences of scores between the pre-trial and post-trial phases in the two groups. We considered pre-trial scores as the dependent variable and trial intervention as the fixed factor. Also, partial eta squared (η^2^) was employed to explore effect sizes. As indicated by Cohen (1998), we defined the effect sizes as small (η^2^ = 0.01), medium (η^2^ = 0.06), and large (η^2^ = 0.14) effects (Cohen, 1998).

### Protocol Registration

This study was registered in the Iranian Registry of Clinical Trials, with clinical trial identifier IRCT 20110228005931N5 and URL https://en.irct.ir/trial/33468.

## Results

### Sample Characteristics

Table 2 presents the characteristics of the study population. Most women were 24–25 years old and had a high-school education level. There were no significant differences between psychotherapy and PUC at baseline in terms of the demographic characteristics. The primary outcomes have been presented in Table 3.

**TABLE 2:** Characteristics of the participants.

Variable	Intervention (*n* = 35)	Control (*n* = 35)	*P*-value
Age (years), mean(*SD*)	25.45 (5.26)	24.47 (4.27)	0.39
Education (years), mean(*SD*)	13.03 (2.660)	12.96 (2.78)	0.91
Gestational age (weeks), mean(*SD*)	27.60 (1.88)	28.11 (2.02)	0.27
Job of women, *N*(%)			0.33
Unemployee	28(82.4)	24 (72.7)	
Employee	6 (17.6)	9 (27.3)	
Job of husband, *N*(%)			0.79
Unemployee	7 (20.6)	6 (18.2)	
Employee	27(79.4)	27(81.8)	
Number of pregnancies, *N*(%)			0.94
1	25(71.4)	24(70.6)	
≤2	10(28.6)	10(29.4)	
History of abortion, *N*(%)			0.96
Yes	5(14.3)	5(14.7)	
No	20(85.7)	29(85.3)	

**TABLE 3:** Between-group differences at post-treatment (T1) and pre-treatment (T0) and effect sizes for assessments.

Outcomes	Intervention (*n* = 35)		Control (*n* = 34)		Differences (T1 − T0)^a^		*B* group effect	*P*	η^2^*
	T0 Mean(*SD*)	T1 Mean(*SD*)	T0 Mean(*SD*)	T1 Mean(*SD*)	Intervention Mean(*SD*)	Control Mean(*SD*)			
W-DEQ									
Lack of self-efficacy	15.54(7.05)	10.48(7.27)	15.11(8.9)	17.82(7.75)	−5.17(8.30)	2.82(5.24)	−7.57	<0.001	0.29
Fear	11.68(5.25)	8.25(5.52)	11.58(6.05)	12.67(5.17)	−3.45(6.10)	1.11(3.51)	−4.47	<0.001	0.22
Negative appraisal	11.68(5.25)	8.25(5.52)	4.58(3.16)	5.03(2.91)	−2.23(3.78)	0.62(2.00)	−1.97	0.001	0.15
Lack of positive anticipation	4.45(2.48)	3.28(3.28)	3.85(2.54)	4.55(3.06)	−1.40(2.61)	0.94(2.48)	−1.75	0.006	0.11
Concerns for the child	2.94(2.48)	2.25(2.68)	3.41(2.93)	3.91(2.58)	−0.54(3.02)	0.35(1.30)	−1.37	0.008	0.10
Loneliness	14.31(8.01)	9.40(7.60)	13.41(7.97)	14.58(7.20)	−5.16(8.61)	1.43(4.30)	−5.70	<0.001	0.19
Total scores	56.22(23.01)	38.38(24.21)	53.82(27.36)	60.58(24.12)	−18.51(28.10)	7.43(15.32)	−23.54	<0.001	0.27
NuPDQ									
Medical and financial problems	2.54(1.46)	1.37(1.41)	1.94(1.53)	2.11(1.68)	−1.36(1.58)	0.37(0.99)	−1.13	<0.001	0.18
Parenting	3.20(1.58)	2.48(1.86)	2.64(1.64)	2.97(1.71)	−0.87(1.90)	0.49(1.29)	−0.82	0.03	0.07
Infant health	2.17(1.54)	1.25(1.44)	1.58(1.28)	1.82(1.11)	−1.05(1.54)	0.38(0.95)	−0.86	0.002	0.14
Physical symptoms	1.77(1.76)	1.65(1.89)	1.58(1.53)	1.76(1.28)	−0.17(1.43)	0.23(1.21)	−0.23	0.438	0.01
Labor and delivery	2.25(1.12)	1.28(1.15)	1.70(1.33)	2.03(1.33)	−1.12(1.33)	0.49(0.97)	−1.05	<0.001	0.20
Total score	12.57(5.29)	8.51(6.26)	10(5.55)	11.41(5.24)	−4.85(5.70)	2.23(4.36)	−4.51	<0.001	0.19
State-anxiety	40.28(13.45)	30.42(16.12)	40.02(10.27)	42.73(8.41)	−9.91(50.87)	2.76(7.09)	−12.42	<0.001	0.22
Self-efficacy	94.31(33.92)	94.74(53.90)	87.23(40.34)	90.44(36.23)	−2.03(17.81)	5.74(21.81)	−0.70	0.94	0.001

^a^Estimated means obtained from analysis of covariance (ANCOVA) after controlling baseline. *Partial eta squared. W-DEQ, Wijma Delivery Expectancy/Experience Questionnaire; NuPDQ, Prenatal Distress Questionnaire.

Table 4 shows the clinical changes of the participants in the scores of the FOC and pregnancy stress. At the baseline, 47 of 59 subjects (about 80%) had FOC based on the cutoff score of W-DEQ >60. Also, 49 of 59 women (about 83%) had pregnancy stress based on the cutoff score of NuPDQ yes >16. The result of chi-square tests revealed that improvement of FOC with the cutoff score of W-DEQ ≤ 60 in MI psychotherapy was significantly greater than that in the PUC group (62.9% vs. 25.5%, *p* = 0.002). Also, improvement of pregnancy stress with a score of W-DEQ ≤ 16 in MI psychotherapy was significantly greater than that in the PUC group (51.4% vs. 23.5%, *p* = 0.016).

**TABLE 4:** Clinical changes in scores of fear of childbirth and pregnancy stress in two groups of participants from pre-trial to post-trial.

Variable scores	MI psychotherapy		Prenatal usual care (PUC)		***P-value
	No *N* (%)	Yes *N* (%)	No *N* (%)	Yes *N* (%)	
Fear of childbirth (FOC)*					
Pre-trial	10 (28.6)	25 (71.4)	12 (35.3)	22 (64.7)	0.367
Post-trial	22 (62.9)	13 (37.1)	9.0 (25.5)	25 (73.6)	0.002
Pregnancy stress (NuPDQ)**					
Pre-trial	8.0 (22.9)	27 (71.1)	12 (35.3)	22 (64.7)	0.191
Post-trial	18 (51.4)	17 (48.6)	8.0 (23.5)	26 (76.5)	0.016

*Cutoff score for FOC: W-DEQ yes > 60, no ≤ 60. **Cutoff score for pregnancy stress: NuPDQ score yes > 16, no ≤ 16. ***Results from chi-square tests.

### Primary Outcomes

#### Fear of Childbirth

The total score of W-DEQ declined more considerably in the psychotherapy group than in the TAU group between pre-trial (T0) and post-trial (T1), with a large effect size (*B* = −23.54, *p* = <0.001, η^2^ = 0.27). Also, scores of the six subscales of W-DEQ diminished more substantially in psychotherapy than in PUC, including lack of self-efficacy with a large effect size (*B* = −7.57, *P* = <0.001, η^2^ = 0.29), fears with a large effect size (*B* = −4.47, *P* = <0.001, η^2^ = 0.22), negative appraisal with a large effect size (*B* = −1.97, *P* = <0.001, η^2^ = 0.15), lack of positive anticipation with a large effect size (*B* = −1.75, *P* = 0.006, η^2^ = 0.11), concerns for the child with a large effect size (*B* = −1.37, *P* = 0.008, η^2^ = 010), and loneliness with a large effect size (*B* = −5.70, *P* = <0.001, η^2^ = 0.19).

#### General Anxiety

The psychotherapy reduced the scores of state anxiety more considerably than TAU did, with a medium effect size (*B* = −12.42, *P* = <0.001, η^2^ = 22).

#### Pregnancy-Specific Stress

The total score of PSS (NuPDQ) decreased more considerably in the psychotherapy than in the TAU group between pre-trial (T0) and post-trial (T1), with a medium effect size (*B* = −4.51, *p* = < 0.001, η^2^ = 19). Also, the scores of four subscales of NuPDQ dropped more remarkably in the IMP group than in PUC, including concern about medical and financial problems with a large effect size (*B* = −1.13, *P* = < 0.001, η^2^ = 0.18), concern about parenting with a medium effect size (*B* = −0.82, *P* = 0.03, η^2^ = 0.07), concern about infantile health with a large effect size (*B* = −0.86, *P* = 0.002, η^2^ = 0.14), and concern about labor and delivery with a large effect size (*B* = −1.05, *P* = <0.001, η^2^ = 0.20). However, concern about physical symptoms did not differ between the psychotherapy and PUC groups (*P* < 0.05).

#### Childbirth Self-Efficacy

The scores of self-efficacy and concern about physical symptoms did not differ between the psychotherapy and PUC groups (*P* < 0.05).

### Secondary Outcomes

#### Subjective Clinical Improvement of FOC

For the women who attended psychotherapy, the mean of FOC diminished subjectively from pre-trial, 6.10 ± 2.15, to post-trial, 2.73 ± 1.88 (*t* = 8.883, *p* < 0.001).

#### Treatment Compliance

The mean number of psychotherapy sessions attended was 3.8 (*SD* 0.98). Of the 35 women of the psychotherapy group, 23 attended four to five sessions of the treatment (65.7% compliance), with 85.7% providing post-treatment data. Of the 35 women of the PUC group, 34 provided post-treatment data (97.1%). Also, the psychotherapist rated 82.8% of the proposed assignments of the participants as good quality.

#### Treatment Satisfaction

The mean of participants’ satisfaction with the program was very high (4.82 ± 0.52). Indeed, most of them were very satisfied with the sessions (29/35, 82.8%).

## Discussion

The aim of the current study was to examine the efficacy of psychotherapy on FOC, pregnancy stress, and self-efficacy. The psychotherapy led to moderate to large improvements in the scores of FOC, PSS, and general anxiety.

At post-trial, the psychotherapy reduced the W-DEQ and its subscales with a large effect size, greater than that of PUC. As this study has been the first RCT to report the effectiveness of psychotherapy on the fear of pregnant women, we compared these results with other psychotherapies. Consistent with the present study, a study concluded that modified midwifery care, full-time midwives supporting pregnancy, as well as labor and childbirth improved FOC as well as pregnant women’s satisfaction (Hildingsson et al., 2018). Another study reported that 2 h delivery training by midwives significantly reduced the FOC (Haapio et al., 2017). Also, 18 h of a mindfulness-based maternity preparedness program improved fear and labor pain, as well as self-efficacy, in pregnant women (Duncan et al., 2017). A study reported that group relaxation training reduced the total FOC scores by 10.30 (Rouhe et al., 2015). Although other counseling methods reduced the FOC, there have been differences in the number of sessions, number of participants, and methodology between the present study and past research.

We found that psychotherapy decreased PSS more than PUC did, with a large effect size. Although we did not find any published study to report the effectiveness of psychotherapy on PSS, the effect of other psychological interventions has been reported. Matvienko-Sikar and Dockray (2017) emphasized that positive psychological intervention reduced the PSS of 46 Australian pregnant women. Chan (2015) improved pregnancy stress and pregnancy outcomes with six sessions of mindfulness mediation intervention in 123 Chinese pregnant women. Richter et al. (2012) reported the early effect of cognitive behavioral group intervention on daily stress and cortisol in pregnant women with symptoms of stress, anxiety, and depression (Richter et al., 2012). Also, a previous study reported the effect of CBT on improvement of PSS and pregnancy complications (Asghari et al., 2016).

In the present study, the MI psychotherapy reduced the level of general anxiety of pregnant women with a large effect size compared to TAU. In line with these findings, some previous studies have found that psychological interventions could reduce general anxiety in pregnant women. The mindfulness group therapy program, with six sessions of 2 h once a week, reduced the score of state anxiety (Woolhouse et al., 2014). In another study, the rate of general anxiety diminished moderately with six sessions of mindfulness (Guardino et al., 2014). A study reported the reduction of general pregnancy anxiety in a three-session intervention by a midwife (Andaroun et al., 2018). It is supposed that different reductions of general anxiety in various studies and the number of sessions may be related to the type of the therapy, the duration of the therapy, and the person conducting the psychotherapy.

Note that in this study, the group psychotherapy did not affect the self-efficacy of the pregnant women. In contrast with this result, there are some reports on the effectiveness of some interventions on the self-efficacy of pregnant women. The effectiveness of prenatal educational intervention (Lp et al., 2009), online prenatal care (Tsai et al., 2018), a mindfulness program (Pan et al., 2019), and a maternal preparedness program (Howarth and Swain, 2019) on enhancing self-efficacy in pregnant women has been reported in previous studies. Also, a study reported that MI enhanced self-efficacy and promoted weight loss in overweight and obese adolescents (Miller and Rollnick, 2013).

The question now is why psychotherapy did not improve the self-efficacy and physical symptoms. Although the response is not clear, some hypotheses can be proposed. First, MI is a client-centered treatment. Thus, the therapist would focus on items considered by the patient as the main FOC or the main cause of anxiety in pregnancy. Previous studies argued that fear of pain and its tolerance, fear of injury to the infant, and fear of complications of vaginal delivery were the most important reasons for the fear of vaginal delivery (Omidvar et al., 2018). Hence, some variables that did not change in the psychotherapy may have been the items which were not very important for the clients during the 5 weeks of psychotherapy. Definitely, further research should investigate why clients do not focus on their other concerns. Further, the short duration of the therapy (five sessions) could justify why some stresses such as physical symptoms remained addressed.

Now, we should explain how group psychotherapy led to moderate to high improvement in FOC, general stress, and PSS. Although the exact mechanism of the psychotherapy on reducing fears or anxiety is not yet clear, some hypotheses can be propounded. First, the nature of MI psychotherapy is a collaborative, goal-oriented therapy of communication with a particular attention to change. An MI psychotherapy session involves partnership with the client, acceptance of the client, promotion of the client’s welfare, and prioritization of her needs, as well as stimulation of the client/patient’s own motivation (Miller and Rollnick, 2013). In addition, just as therapists help clients resolve their ambivalence about behavioral change (FOC and anxiety), they also encourage their motivation for and commitment to that change (Miller and Rollnick, 2013). Secondly, the nature of the FOC and pregnancy stress is related to ambivalences. Thus, the congruence between the nature of FOC plus pregnancy stress and the nature of the methods of MI psychotherapy, increasing motivation for resolving the ambivalences and commitment to the intervention, may have contributed to the effect of MI psychotherapy on improving FOC and pregnancy stress in 5 weeks post-trial. The therapist helps reduce the fear by understanding and fueling pregnant women’s motivation, asking open-ended questions about their concerns and fears, actively listening and providing the information they need, and then empowering them (O’Connell et al., 2017). Also, the therapist encourages the client to obtain sufficient information on how to cope with the pain of labor and delivery, to confront the client with her fears, in particular, the fear of vaginal injury (Ghazaie et al., 2019). The therapist also motivates the client to prioritize her values and lists the benefits as well as disadvantages of vaginal childbirth and cesarean section. Also, the therapist helps clients identify tempting risky situations of childbirth to help them choose the appropriate childbirth they would prefer (Rosengren, 2013).

### Limitations

This study had some limitations that should be considered when generalizing the results. First, the evaluation of FOC, pregnancy stress, and self-efficacy of pregnant women was performed through self-report questionnaires. Use of clinical interviews could be a better indicator for diagnosis or changes in fear and anxiety. It is suggested that in the future, clinical interviewing be used to diagnose the severity of FOC, stress, or women’s self-efficacy. Secondly, this study did not report the sustainability of the effects of MI psychotherapy, as the pregnant women were not followed up until the delivery. Although pregnant women were contacted by the principal investigator via smartphone after the sessions, and they were verbally informed about their condition until the delivery, unfortunately, we were not able to convince them to complete the fear and stress questionnaires again. Also, it would be better to assess the experience of fear during childbirth or self-efficacy during delivery. This study recommends that future studies measure the effectiveness of psychotherapy on the actual experience of the fear of delivery or self-efficacy near delivery, during delivery, and after delivery. Further, this study was conducted on low-risk women, so its generalization to high-risk pregnancies is another limitation. High-risk pregnancies may cause more pregnancy stresses, and women are likely to be more afraid of normal delivery. Therefore, a future RCT is recommended for assessing the effect of psychotherapy in women with high-risk pregnancies. Finally, our study evaluated the effect of MI psychotherapy on changes of FOC in pregnant women. Further research is required to assess the effect of psychotherapy in the treatment of women with the diagnosis of FOC.

## Conclusion

To the best of our knowledge, this study was the first RCT for the efficacy of MI psychotherapy on FOC and pregnancy stress. We found that adding 5 weeks of group MI psychotherapy to PUC could be considered as an adjunctive care option for reducing FOC, PSS, and general anxiety in pregnant women in the third trimester. Further research including an economic evaluation of adding psychotherapy to PUC would be useful. The findings suggested that psychotherapy could be proposed for improving the PUC of women in the third trimester of pregnancy.

## Data Availability Statement

The raw data supporting the conclusions of this article will be made available by the authors, without undue reservation, to any qualified researcher.

## Ethics Statement

The study was approved by the Ethic Committee of Babol University of Medical Sciences (IR.MUBABOL HRLREC. 1397.116). Written informed consent was obtained directly from the participants.

## Author Contributions

MF was the principal investigator of this study. SA contributed development of the project, gathering the data, and writing the manuscript. MD contributed to the development of the project. FB was involved in managing the project. MC and HG performed the statistical analyses. All authors contributed to the drafting of this paper and approved the final manuscript.

## Conflict of Interest

The authors declare that the research was conducted in the absence of any commercial or financial relationships that could be construed as a potential conflict of interest.

## Footnotes

1 www.random.org
